# Goldilocks Principle: Preference for Change in Breast Size in Breast Cancer Reconstruction Patients

**DOI:** 10.3389/fpsyg.2021.702816

**Published:** 2021-09-03

**Authors:** Krista M. Nicklaus, Thao Bui, Mary Catherine Bordes, Jun Liu, Deepti Chopra, Aubri S. Hoffman, Gregory P. Reece, Summer E. Hanson, Fatima A. Merchant, Mia K. Markey

**Affiliations:** ^1^Department of Biomedical Engineering, The University of Texas at Austin, Austin, TX, United States; ^2^Department of Plastic Surgery, The University of Texas MD Anderson Cancer Center, Houston, TX, United States; ^3^Department of Engineering Technology, University of Houston, Houston, TX, United States; ^4^Department of Psychiatry, The University of Texas MD Anderson Cancer Center, Houston, TX, United States; ^5^Department of Gynecologic Oncology and Reproductive Medicine, The University of Texas MD Anderson Cancer Center, Houston, TX, United States; ^6^Section of Plastic and Reconstructive Surgery, University of Chicago Medicine and Biological Sciences, Chicago, IL, United States; ^7^Department of Imaging Physics, The University of Texas MD Anderson Cancer Center, Houston, TX, United States

**Keywords:** breast reconstruction, breast cancer, body image, quality of life, outcome assessment, health care

## Abstract

Patients’ preferences regarding changing or maintaining their breast size after mastectomy and reconstruction are important but understudied determinants of post-surgical satisfaction and quality of life. The goal of this study was to identify factors associated with preferences for changing or maintaining breast size for women undergoing breast reconstruction at The University of Texas MD Anderson Cancer Center in the United States from 2011 to 2014. The average age of participants was 45.7 ± 9.1 years. At baseline, mean average breast volumes were 755.7 ± 328.4 mL for all women (*n* = 48), 492.3 mL ± 209.3 for 13 women who preferred to be “bigger than now,” 799.2 mL ± 320.9 for 25 women who preferred to remain “about the same,” and 989.3 mL ± 253.1 for 10 women who preferred “smaller than now.” Among the 23 women who preferred to change their breast size, 19 desired to shift toward the mean. Women with the smallest and largest 20% of baseline breast size were more likely to desire a change toward the mean (*p* = 0.006). Multinomial logistic regression models found average breast volume and satisfaction with breast size to be the most important factors associated with preferences for changing or maintaining breast size for women undergoing breast reconstruction. This study provides preliminary evidence for a “Goldilocks principle” in women’s preferences for breast size change in the context of breast reconstruction, and identifies hypotheses for future studies of the associations among preference for change in breast size, preference achievement, and post-reconstruction body image.

## Introduction

The purpose of breast reconstruction is to recreate the look of breast mounds when clothed. Each patient has unique expectations and preferences for their surgical and aesthetic outcomes. Uhlmann et al. (1984) define patient expectations as “anticipations that given events are likely to occur” and desires, or preferences, as “a perception that a given event is wanted.” Patients’ expectations of their surgical outcomes are often formed by information they’ve gathered about breast reconstruction from their providers, social contacts, etc., as well as their intuition. Patients’ preferences for how their body will look like after surgery are formed by each patient’s individual experience with their body and psychological well-being, separate from their preferences regarding treatment type. Patients’ expectations and preferences are distinct and may have different impacts on the psychosocial benefits of reconstruction. For example, a patient may prefer that she maintain her pre-surgery breast size but expect her breast size to change due to limitations of the reconstructive process. Knowledge about the importance of patients achieving their breast reconstruction expectations and preferences may inform psychosocial care of breast cancer patients.

Several studies have researched how patient expectations impact satisfaction with breast reconstruction outcomes and quality of life. Flitcroft et al. (2017) conducted a review of 20 studies that researched breast reconstruction patients’ expectations from 1994 to 2017. These studies used a variety of qualitative, quantitative, and mixed methods to assess patients’ expectations of breast reconstruction outcomes. Five studies, which quantified expectations and whether expectations were met, discovered a positive correlation between meeting expectations and satisfaction with outcomes. The review identified the need for consistent methods to capture and measure patient-reported expectations and outcomes. Another study examined how women’s expectations around reconstruction change over time. de Boer et al. (2015) conducted a longitudinal phenomenological study with interviews of 7 women at several time points from before their reconstruction surgery to 1 year after the surgery. The researchers found the participants tended to focus their expectations of surgical outcomes on three aspects: (1) how their body looked, (2) how their body functioned, and (3) how their body felt. Snell et al. (2010) provide an example of how research into expectations can help healthcare providers improve patient education and thus satisfaction. The researchers conducted interviews to study unfulfilled patient expectations about implant-based breast reconstruction, identifying areas where patients lacked information and were unsatisfied with their results. These publications represent some of the ways that expectations have been studied for breast reconstruction patients. These and other studies have shown that women’s expectations of their surgical outcomes affect their outcome and can be mediated by patient education interventions.

There has been limited work, however, on women’s preferences for surgical outcomes. Bailey et al. (2010, 2012) assessed women’s preference for scar location for latissimus dorsi flap reconstruction, and in a separate study, gathered opinions about the most important aesthetic subunit of the breast. Both of these studies used female participants with and without a history of breast cancer or breast surgery. The focus of these studies was primarily to improve surgical planning, and did not include any psychosocial measures. Sun et al. (2014) elicited healthy women’s preferences for five different BREAST-Q outcomes: satisfaction with breasts, psychosocial well-being, chest well-being, abdominal well-being, and sexual well-being for analyzing preference models and decision-making. Several studies assessed the effect of surgical outcomes on women’s psychosocial well-being, but did not take into account women’s preferences for those outcomes (Yip et al., 2015; Teo et al., 2018). The effect of achieving or not achieving patients’ surgical preferences on their satisfaction with reconstruction overall or psychosocial well-being, such as body image, is currently unknown.

A difficulty for both patients and researchers when studying expectations of or preferences for breast reconstruction outcomes is that it is cumbersome to measure the patient’s mental images of possible future states of her body. For example, Mace et al. (2021) analyzed conversations between breast reconstruction patients and their surgeons about breast asymmetry. The patients used a combination of gestures to their body, anecdotes, and descriptions of their emotions to express their concerns about asymmetry, which the surgeon had to interpret to fully understand their concerns. An important surgical outcome that is arguably the easiest for patients to articulate their thoughts about is breast size. There are straightforward ways that a woman can communicate an approximation of her preferred or expected post-reconstruction breast size, such as by bra cup size or by indicating larger, smaller, or about the same size as her pre-reconstruction breast size. In addition, there are established methods to objectively and quantitatively measure breast size, such as breast volume on 3D photography, that may be useful for relating to the patient’s preferences or expectations. For these reasons, this study focused on women’s preferences for a change in their breast size. The goal of this study was to identify factors that are associated with preference for change in breast size for women undergoing breast reconstruction at an American medical institution.

## Materials and Methods

### Patient Population

The study population consisted of women who underwent breast reconstruction at The University of Texas MD Anderson Cancer Center in the United States from 2011 to 2014. As part of an institutionally reviewed research project (IRB #2010-0321) (Reece et al., 2015), medical record, demographic, 3D torso images, and psychosocial health data were collected pre-reconstruction and at 6, 9, 12, 18, and 18+ months after patients’ initial reconstructive procedure. 3D photographs (surface scans) of the patients’ torsos were collected with a customized 3dMDTorso System (3dMD, LLC, Atlanta, GA). All participants provided informed consent.

For this study sample, participants were selected who: underwent reconstruction after a skin-sparing total mastectomy and had complete data, including a baseline stated preference for maintaining/changing breast size, as well as 3D images and Body Image Scale scores collected pre-/post-operatively (i.e., at baseline and after the initial reconstructive surgery).

### Measures and Measurements

At baseline, participants responded to a question that asked for their preferred post-reconstruction breast size, with three possible responses: (1) “About the same size as I am now,” (2) “Bigger than I am now,” and (3) “Smaller than I am now.” For this analysis, participants were therefore grouped by their preference for maintaining/changing their breast size into three groups: About the Same, Bigger than Now, and Smaller than Now. Participants also responded to the Brief Symptom Inventory-18 (BSI-18) (Derogatis, 2000), Appearance Schemas Inventory – Revised (ASI-R) (Cash et al., 2004), Body Image Scale (Hopwood et al., 2001), and BREAST-Q Reconstruction modules (Pusic et al., 2009), which assess levels of psychological distress, investment in their appearance, body image concerns, and satisfaction with breast reconstruction, respectively. Prior studies have validated the measures for cancer patients and demonstrated internal consistency; BSI-18: (Galdón et al., 2008; Grassi et al., 2018; Calderon et al., 2020), ASI-R: (Moreira et al., 2010a; Chua et al., 2015), BIS: (Moreira et al., 2010b; Melissant et al., 2018; Shunmuga Sundaram et al., 2019), BREAST-Q: (Cano et al., 2012; Cohen et al., 2016; Mundy et al., 2017).

Two authors (KN and TB) measured breast volume on the 3D photographs using proprietary software developed by our team (Lee et al., 2011). Ptosis was graded by an experienced reconstructive surgeon (GR) from the clinical photographs on a scale of 0–3, with 0 indicating no ptosis.

### Analyses

Descriptive statistics summarized the distributions of women’s pre-reconstruction breast volumes and their preferences for maintaining/changing breast size (i.e., smaller than now, about the same, and bigger than now). Breast volumes were calculated as the average between both breasts. Based on distributions, we then assessed the rates of women in the smallest or largest 20% of average breast volumes who preferred a “middling” change (i.e., smallest 20% preferring “bigger than now” and largest 20% preferring “smaller than now”), compared to all other participants. As distributions allowed, we also explored correlations across all size (smallest 20%, average 60%, largest 20% volume) and preference (smaller than now, about the same and bigger than now) groups.

We then summarized the distributions of women’s clinical and psychosocial data amongst the preference groups (i.e., smaller than now, about the same, and bigger than now) using box and whisker plots. Univariate analyses assessed differences of variables amongst preference groups. Kruskal-Wallis and Wilcoxon Rank Sum tests with Bonferroni correction were used for continuous data types (e.g., age, breast volume), and Chi-square tests were used for categorical data (e.g., Relationship Status).

Lastly, we used multinomial logistic regression to investigate which factors are associated with breast size preference. The covariates studied were age, body mass index (BMI), relationship status, average breast volume, average ptosis, satisfaction with breast size (1–5 scale with 5 being most satisfied), satisfaction with current weight (1–5 scale with 5 being most satisfied), ASI-R composite score, BSI global score, BIS score BREAST-Q satisfaction with breasts (SWB) module, and BREAST-Q psychosocial well-being (PSWB) module. Covariates with Type III *p*-values of less than 0.2 in univariate analysis were considered candidate variables for model selection. The model fit criteria, Akaike information criterion (AIC), was used to identify the best fit multiple multinomial logistic regression model from an exhaustive search of all combinations of two covariate models, three covariate models, and four covariate models. A maximum of four covariates were used in the model because of the sample size. The Hosmer and Lemeshow test was also used analyze model performance. All analyses were performed in R (R Core Team, 2019) and MATLAB R2019a (Mathworks, MA, United States).

## Results

### Patient Characteristics

Forty-eight patients met the criteria for this study. All patients indicated female as their biological sex. At baseline, the mean age was 46 ± 9 years, mean BMI was 26.7 ± 4.5 kg/m^2^, and 41 (85%) were White. Reconstruction types included 30 (63%) implant, 14 (29%) autologous (TRAM and DIEP), and 4 (8%) implant plus autologous. Thirty-one patients (65%) had reconstruction on both breasts. Supplementary Figure 1 shows the patient sample selection process. Table 1 presents the patient characteristics data.

**TABLE 1:** Patient characteristics.

		Preference for change in breast size		
		
	Overall	About the Same	Bigger than Now	Smaller than Now
N	48	25	13	10
Age, Years (Mean ± SD)	45.7 ± 9.1	47.6 ± 9.2	43.8 ± 9.3	43.4 ± 8.5
BMI (Mean ± SD)	26.7 ± 4.5	26.7 ± 3.9	24.0 ± 3.6	29.7 ± 5.2
**Race (N)**				
White	41 (85%)	23 (92%)	10 (77%)	8 (80%)
Black or African American	3 (6%)	0	1 (8%)	2 (20%)
Asian	1 (2%)	1 (4%)	0	0
Other	1 (2%)	1 (4%)	0	0
Did Not Specify	2 (5%)	0	2 (15%)	0
**Ethnicity (N)**				
Non-Hispanic	38 (79%)	22 (88%)	11 (85%)	5 (50%)
Hispanic	9 (19%)	3 (12%)	2 (15%)	4 (40%)
Did Not Specify	1 (2%)	0	0	1 (10%)
**Reconstruction Type (N)**				
Implant	30 (63%)	15 (60%)	7 (54%)	8 (80%)
Autologous	14 (29%)	9 (36%)	3 (23%)	2 (20%)
Mixed (Implant/Latissimus Dorsi)	4 (8%)	1 (4%)	3 (23%)	0
Post-reconstruction Radiation Therapy (N)	7	3	3	1
Bilateral Procedure (N, Ref: Unilateral Procedure)	31	15	10	6
Symmetry Procedure if Unilateral (N, Ref: No symmetry procedure)	12	5	3	4
**Post-operative Study Visit Time Point (N)**				
6 Months	1	0	1	0
9 Months	5	2	0	3
12 Months	4	3	0	1
18 Months	21	13	7	1
18+ Months	17	7	5	5
Pre-operative BIS Score (Median, Range)	4, 0–28	1, 0–28	5, 0–16	7.5, 0–24
Post-operative BIS Score (Median, Range)	5, 0–29	7, 0–27	3, 0–21	9.5, 0–29

### Descriptive Statistics

**FIGURE 1:**
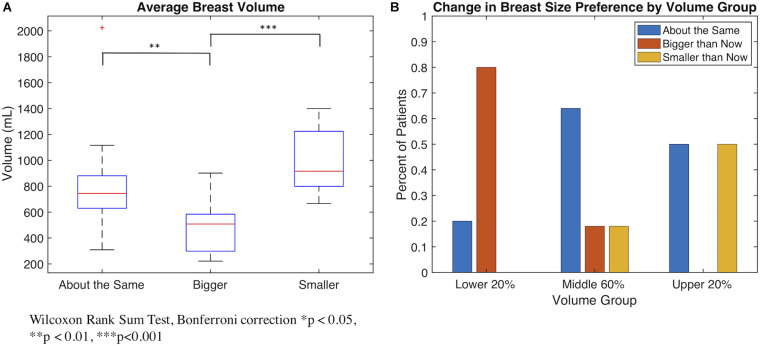
**(A)** Boxplot demonstrating the average breast volume of patients distributed by patients’ preference for change in breast size. Patients who desired a change in breast size tend to have breast volumes further away from the mean than patients who preferred to remain about the same size. **(B)** Bar plot demonstrating the preference for change in breast size of patients in different pre-operative volume groups (lower 20% of volume, middle 60%, and upper 20%).

At baseline, the mean average breast volume was 755.7 ± 328.4 mL for all women. Overall, 23 women stated a baseline preference for changing their breast size – 13 preferred “bigger than I am now” and 10 preferred “smaller than I am now” – and 25 preferred “about the same size as I am now.” The mean average breast volume at baseline was 492.3 mL ± 209.3 for the “bigger than I am now” group; 989.3 mL ± 253.1 for the “smaller than I am now” group; and 799.2 mL ± 320.9 for the “about the same” group. Breast volumes were significantly different between the “bigger than now” group and the other two preference groups (compared to the “about the same”: *p* = 0.002; compared to the “smaller than now” group: *p* < 0.0001, based on the Wilcoxon rank sum test with Bonferroni correction) (Figure 1A). Among the 23 women who preferred a change in breast size, 19 (83%) preferred to shift toward the mean, and 4 (17%) preferred to shift further away from the mean.

Given that the majority of women preferred to shift toward the mean, we conducted a subgroup analysis of the 10 women with the smallest 20% of breasts (<221.54–520.08 mL) and 10 women with the largest 20% of breasts (>963.27–2024.55 mL), compared with the 28 women with average breast sizes (mean average breast volume of 728.03 mL). Among the 20 women with the smallest/largest breast sizes, 13 (65%) preferred to change their breast size (8 preferred “bigger than I am now” and 5 preferred “smaller than I am now”) and 7 preferred “about the same size as I am now” (Figure 1B). Among these 13 women who preferred a change in breast size, all 13 (100%) preferred to shift toward the mean, and 0 (0%) preferred to shift further away from the mean. Compared to all others (*n* = 28), the 20 women with the smallest or largest breasts were more likely to express a preference for changing their breast size toward the mean (65% versus 21%, respectively, *p* = 0.006).

For the 60% of women with more average breast sizes, 5 (18%) preferred “bigger than I am now,” 18 (64%) preferred “about the same size as I am now,” and 5 (18%) preferred “smaller than I am now.” Among the 10 (36%) women who preferred a change in breast size, 6 (60%) preferred to shift toward the mean, and 4 (4%) preferred to shift further away from the mean. Compared to the group of women with the smallest or largest breasts, differences were observed in their likelihood of preferring to change their breast size (*p* = 0.08) or to shift toward the mean (*p* = 0.006).

**FIGURE 2:**
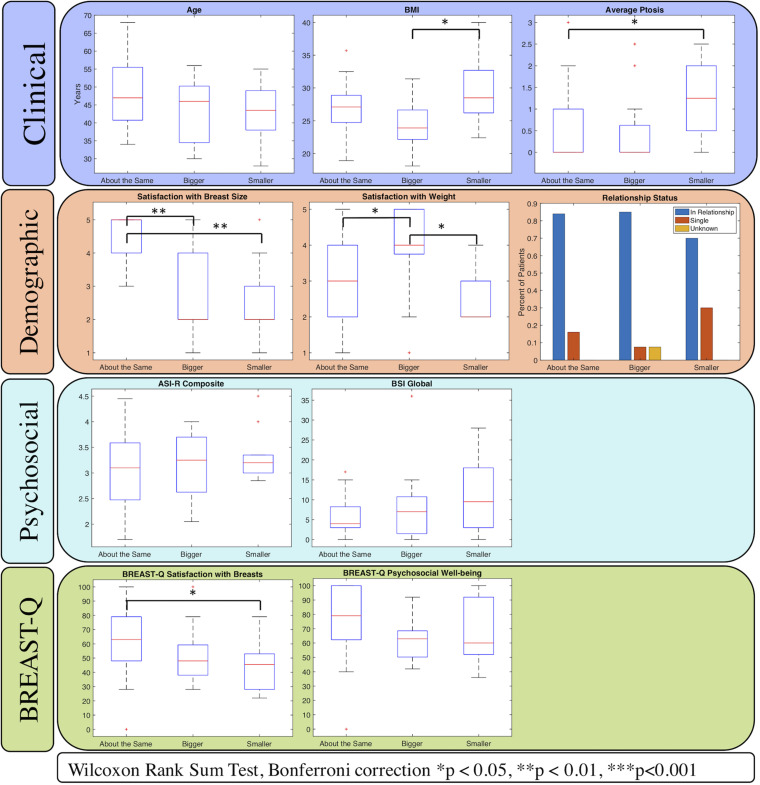
Relationships between patients grouped by their preferences for change in breast size with potential covariates. Four categories of variables were selected as potential covariates: clinical, demographic, psychosocial, and the BREAST-Q. Variables with significant differences amongst the groups are marked with asterisks. Average breast volume (Clinical variable) is depicted in Figure 1.

Figure 2 depicts the distribution of relevant data amongst the three preference groups. In addition to breast volume, two other clinical variables, BMI and average ptosis, had significant differences amongst preference groups. BMI varied significantly between the “bigger than now” group and “smaller than now” group (*p* = 0.036, Wilcoxon rank sum test with Bonferroni correction). The median ptosis for both “about the same” and “smaller than now” groups was 0, compared to 1.25 for the “smaller than now” group (*p* = 0.031, Kruskal-Wallis test).

Three psychosocial variables differed amongst preference groups to the point of statistical significance. Women who preferred a change in breast size (“bigger than now” and “smaller than now” groups) were significantly less satisfied with their baseline breast size than the women who preferred to remain their current size (*p* < 0.001, Kruskal-Wallis test). Satisfaction with weight was also significantly less for women in the “smaller than now” group than the other two groups (*p* = 0.013, Kruskal-Wallis test). The BREAST-Q Satisfaction with Breasts module also revealed less satisfaction amongst the “bigger than now” and “smaller than now” patients compared to “about the same” patients (*p* = 0.016, Kruskal-Wallis test). Body image concerns, appearance investment, and depression levels did not significantly differ amongst groups. Supplementary Table 1 contains the complete statistical comparisons amongst preference groups.

### Univariate Analyses

We investigated pre-operative age, body mass index (BMI), relationship status, average breast volume, average ptosis, satisfaction with breast size (1–5 scale with 5 being most satisfied), satisfaction with current weight (1–5 scale with 5 being most satisfied), ASI-R composite score, BSI global score, BREAST-Q satisfaction with breasts (SWB) module, and BREAST-Q psychosocial well-being (PSWB) module as covariates that may be associated with breast size preference. Univariate multinomial logistic regression model results are presented in Supplementary Table 2. The “about the same” preference group was used as the reference group in all multinomial logistic models. Eight variables had Type III *p*-values less than 0.2: BMI, average breast volume, average ptosis, satisfaction with breast size, satisfaction with weight, BREAST-Q SWB, BREAST-Q PSWB, and BSI. These factors were included in the feature selection process for the multivariable model.

### Multivariable Analyses

Based on lowest AIC, the most statistically significant model was the two-covariate model, which showed that average breast volume and satisfaction with breast size were significantly associated with breast size preference. The model results are presented in Table 2. The lowest AIC three-covariate model included variables average breast volume, satisfaction with breast size, and BSI. The lowest AIC four-covariate model included variables average breast volume, satisfaction with breast size, BSI, and BREAST-Q PSWB (Supplementary Tables 3, 4). In all three models, average breast volume and satisfaction with breast size are both significantly associated with choosing “bigger than now” versus “about the same.” Satisfaction with breast size is also significantly associated with choosing “smaller than now” versus “about the same.” While not significant, larger BSI scores (indicating more anxiety and depression) are associated with choosing to change one’s breast size. The results are mixed for BREAST-Q PSWB (higher score indicates better psychosocial well-being) between the two preferences for change.

**TABLE 2:** Two-covariate Multivariable Multinomial Logistic Regression Model.

Variable	Preference	Odds ratio (95% CI)	*P* value	Type III *P* value
Average Breast Volume	BTN	0.994 (0.989–0.998)	0.009	<0.001
Average Breast Volume	STN	1.002 (0.999–1.005)	0.165	
Satisfaction with Breast Size	BTN	0.345 (0.154–0.770)	0.009	<0.001
Satisfaction with Breast Size	STN	0.180 (0.065–0.504)	0.001	

*Reference preference group: About the Same.*

*BTN: Bigger than Now Preference Group, STN: Smaller than Now Preference Group.*

*AIC: 67.78, Hosmer-Lemeshow Test: *p* = 0.05.*

## Discussion

Our study suggests that a Goldilocks principle (i.e., not too big or too small) applies in the preferences for change in breast size for breast reconstruction patients. Women who wanted to increase their breast size had smaller breasts pre-operatively, less satisfaction with their pre-operative breast size, and more satisfaction with their pre-operative weight. Those who preferred to be “smaller than now” had larger breasts pre-operatively and less satisfaction with both their breast size and pre-operative weight. The patients who wanted their post-reconstruction breast size to be “about the same” were very satisfied with their pre-operative breast size but had a larger range of satisfaction with their pre-operative weight. The results are consistent with a previous study of healthy Australian women that reported a correlation between larger breast size and increased likelihood of desiring a change in breast size, as well as greater body and breast dissatisfaction (Spencer et al., 2020). There were no statistically significant differences amongst the groups in age, appearance investment, or pre-operative psychosocial health. Reconstruction type was also similarly distributed within each preference group. The “about the same” group underwent the most autologous procedures, possibly since autologous reconstruction requires enough available tissue at the donor site to reconstruct the new breast but a larger BMI can introduce more risk factors for these more invasive surgeries.

Multinomial logistic regression provided additional evidence for this Goldilocks principle. From Table 1, patients with smaller breast volumes are significantly more likely to choose “bigger than now” than “about the same.” Patients with larger breast volumes tend to choose “smaller than now” more so than “about the same.” The regression analysis also highlighted the importance of satisfaction with breast size. Greater dissatisfaction with ones’ breast size was indicative of a preference for change. Patients’ feelings about their breast size after reconstruction appears to be driven mostly by their prior feelings and experiences about their breast size. In our study, psychological distress was not a significant factor, but our sample included only a few patients experiencing psychological distress. For patients experiencing more psychological distress as they undergo cancer care, their emotional state may serve to amplify the desire to change their breast size in order to try to alleviate negative emotions. Acute distress may also hinder patients from accurately stating their desires.

An interesting area of future research would be to investigate whether reconstruction patients actually achieve their preferred breast size. Consider a plausible but admittedly arbitrary cut-off of 20% on the average volume percent change to discretize breast size change, where average volume percent change is calculated by the average of $\frac{P\,o\,s\,t\,o\,p\,V\,o\,l\,u\,m\,e-P\,r\,e\,o\,p\,V\,o\,l\,u\,m\,e}{P\,r\,e\,o\,p\,V\,o\,l\,u\,m\,e}$ for each breast. Applying this simple definition to our sample, about half of the patients in the “bigger than now” group, 64% of the women in the “about the same” group, and 80% in the “smaller than now” group achieved their preference (Supplementary Figure 2). However, we note that such a definition of preference attainment does not take into account the patient’s opinion about whether her final breast size matches her preference. For example, this simple definition would consider a patient who wanted to be bigger and who’s breast size increased by 25% to have achieved her preferred breast size, but it is possible that she actually wanted to be 50% larger. We emphasize that future studies of preference achievement for change in breast size should carefully consider the trade-offs between different approaches to measuring achievement.

Future work on the psychosocial impacts of holding a particular preference and/or preference achievement could inform how patients and providers make decisions about breast reconstruction. For example, we see an interesting trend in our sample for the “bigger than now” group versus the other preference groups in our sample (Supplementary Figure 2). 77% of the “bigger than now” patients reported a positive change in their body image, compared to only 11% of the other two groups. A possible explanation of this result is that the body image concerns of the “bigger than now” patients are focused more around their breasts than other aspects of their bodies. This is supported by their high satisfaction with weight but low satisfaction with breast size. After reconstruction, their body image concerns around their breast size are alleviated. Patients in the “about the same” and “smaller than now” groups report less satisfaction with weight, and those body image concerns are more likely to persist after reconstruction. For some women, when their breast size is changed, they may develop more concerns about other areas of their body. In addition, reconstruction of larger breasts is technically challenging from a surgical perspective and so tends to result in poorer outcomes. Prior studies have found larger breast size (mastectomy weight) to be a risk factor for reconstruction complications (Woo et al., 2016). New body image concerns may also arise around other breast aesthetic factors after reconstruction surgery, such as symmetry, scarring, and shape.

There is a considerable body of prior work on patients’ expectations or lack of expectations about their reconstruction outcomes (Snell et al., 2010; de Boer et al., 2015; Flitcroft et al., 2017). While expectations are not consistently measured or defined (Flitcroft et al., 2017), they are often framed around whether patients have received enough accurate information to have realistic expectations about their results. A unique aspect of this study is our focus on *preferences* about physical appearance, as opposed to expectations. For example, a patient may prefer that her breasts remain about the same size as they are now, but also expect that her breasts will be smaller after surgery because her care team has explained the limitations of the procedure. Prior research suggests that meeting expectations can be an important factor for patient satisfaction and some psychosocial well-being outcomes (Flitcroft et al., 2017). Our study demonstrates the need for more research on the potential role that patient preferences and preference attainment plays in the psychosocial outcomes of breast reconstruction.

The primary limitation of this study is limited sample size. A larger, more diverse study population may reveal different trends or stronger statistical evidence to consider patients’ preferences as well as their expectations.

In conclusion, preliminary evidence is demonstrated for a Goldilocks principle in preferences for change in breast size for women undergoing breast reconstruction. Women with breast sizes on both the lower and upper range expressed greater dissatisfaction with their current (pre-operative) size and preferred a change toward the mean. Regression analysis supported the concept that women’s preferences for breast size after reconstruction are primarily formed by their satisfaction with their current breast size. Acute psychosocial distress resulting from cancer and its treatment may also reinforce a desire for change, or impede expressing their preferences. The results of this study pose opportunities for future research on the psychosocial effects of holding a particular preference and/or achieving one’s preference for change in breast size in the context of breast reconstruction.

## Data Availability Statement

The datasets presented in this article are not readily available because the data used in this study are restricted to protect patient confidentiality and privacy. Access to the data is available only with permission from The University of Texas MD Anderson Cancer Center Institutional Review Board. Requests to access the datasets should be directed to MM.

## Ethics Statement

The studies involving human participants were reviewed and approved by The University of Texas MD Anderson Cancer Center Institutional Review Board. The patients/participants provided their written informed consent to participate in this study.

## Author Contributions

KN performed image measurements, statistical analysis, and wrote the manuscript text. TB contributed to image measurements. MB contributed to data selection. JL reviewed and helped interpreting the statistical analysis. DC, AH, GR, SH, FM, and MM contributed clinical, surgical, and analytical expertise to designing the study questions and interpreting the results. All authors contributed to the manuscript preparation.

## Conflict of Interest

The authors declare that the research was conducted in the absence of any commercial or financial relationships that could be construed as a potential conflict of interest.

## Publisher’s Note

All claims expressed in this article are solely those of the authors and do not necessarily represent those of their affiliated organizations, or those of the publisher, the editors and the reviewers. Any product that may be evaluated in this article, or claim that may be made by its manufacturer, is not guaranteed or endorsed by the publisher.
