# Supplementary figures and images for: Goldilocks Principle: Preference for Change in Breast Size in Breast Cancer Reconstruction Patients
Source: Front Psychol. 2021 Sep 3;12:702816. doi: 10.3389/fpsyg.2021.702816 (PMC8446205; doi:10.3389/fpsyg.2021.702816)

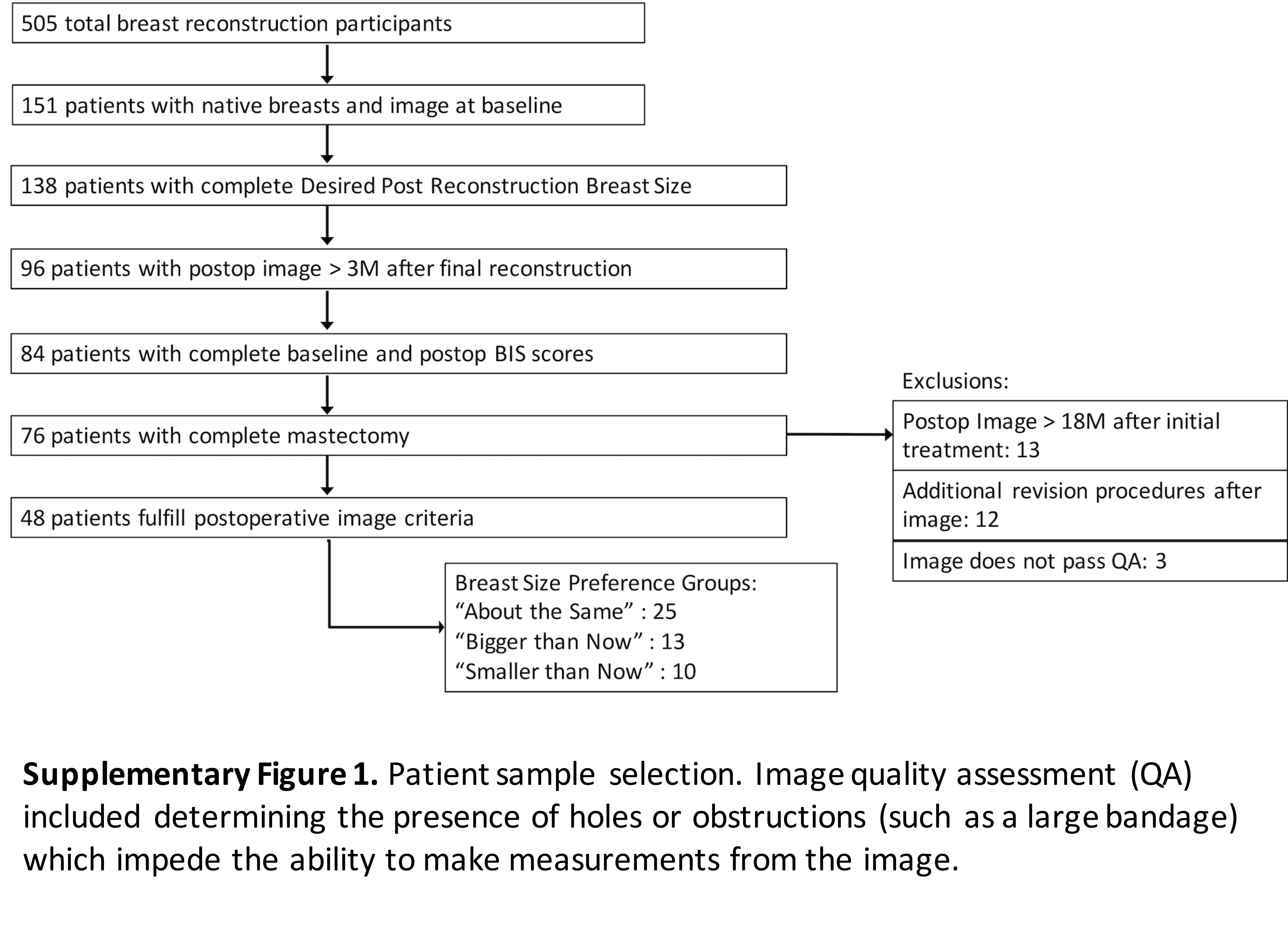

Supplement: Supplementary file 1 [file Image_1.png]
