# Supplementary material for: Goldilocks Principle: Preference for Change in Breast Size in Breast Cancer Reconstruction Patients
Source: Front Psychol. 2021 Sep 3;12:702816. doi: 10.3389/fpsyg.2021.702816 (PMC8446205; doi:10.3389/fpsyg.2021.702816)
Supplement: Supplementary file 3 [file Table_1.docx]

Supplementary Material

# Supplementary Data

Supplementary Material should be uploaded separately on submission. Please include any supplementary data, figures and/or tables. All supplementary files are deposited to FigShare for permanent storage and receive a DOI.

Supplementary material is not typeset so please ensure that all information is clearly presented, the appropriate caption is included in the file and not in the manuscript, and that the style conforms to the rest of the article. To avoid discrepancies between the published article and the supplementary material, please do not add the title, author list, affiliations or correspondence in the supplementary files.

# Supplementary Figures and Tables

For more information on Supplementary Material and for details on the different file types accepted, please see [here](http://home.frontiersin.org/about/author-guidelines#SupplementaryMaterial). Figures, tables, and images will be published under a Creative Commons CC-BY licence and permission must be obtained for use of copyrighted material from other sources (including re-published/adapted/modified/partial figures and images from the internet). It is the responsibility of the authors to acquire the licenses, to follow any citation instructions requested by third-party rights holders, and cover any supplementary charges.

## Supplementary Figures

**Supplementary Figure 1.** Patient sample selection. Image quality assessment (QA) included determining the presence of holes or obstructions (such as a large bandage) which impede the ability to make measurements from the image.

**Supplementary Figure 2.** Change in breast size preference achievement and body image change. The y-axis of this plot demonstrates the average percent volume change (APVC) for each patient, i.e., how much the breasts size increased or decreased after reconstruction. Positive values indicate breast size increased. The x-axis plots BIS Change, i.e., how much the BIS score changed from before reconstruction to after reconstruction. A positive BIS Change indicates body image improved (decrease in body image concerns). The breast size preference groups are differentiated by color and symbol. The horizontal dash lines at +0.2 and -0.2 indicate our chosen tolerance from a change in size. Half (6/13) of the “bigger than now” group (red squares) increased in size, and most (10/13) improved their body image. The “smaller than now” group (yellow asterisks) mostly (8/10) decreased in size, and half experienced a decline in body image. The “about the same” group has 16/25 remained within the chosen tolerance of their native size, and 18/25 experienced a decline in body image.

## Supplementary Tables

Supplementary Table 1. Preference for Change in Breast Size Group Covariate Analyses

| Variable | Preference Group Mean ± SD | Kruskal-Wallis P value | Wilcoxon Rank Sum P value |
| --- | --- | --- | --- |
| Age |  | P = 0.36 | -- |
| About the Same | 47.6 ± 9.2 |  |  |
| Bigger | 43.8 ± 9.3 |  |  |
| Smaller | 43.4 ± 8.5 |  |  |
| BMI |  | P = 0.013 |  |
| About the Same | 26.7 ± 3.9 |  | P = 0.07, vs. Bigger than Now  P = 0.49, vs. Smaller than Now |
| Bigger | 24.0 ± 3.6 |  | P = 0.036, vs. Smaller than Now |
| Smaller | 29.7 ± 5.2 |  |  |
| Relationship Status (N in relationship) |  | P = 0.39† | -- |
| About the Same | 21 (84%) |  |  |
| Bigger | 12 (92%) |  |  |
| Smaller | 7 (70%) |  |  |
| Average Breast Volume |  | P < 0.001 |  |
| About the Same | 799.2 ± 320.9 |  | P = 0.002*, vs. Bigger than Now  P = 0.06, vs. Smaller than Now |
| Bigger | 492.3 ± 209.3 |  | P < 0.001*, vs. Smaller than Now |
| Smaller | 989.3 ± 253.1 |  |  |
| Average Ptosis |  | P = 0.03 |  |
| About the Same | 0.48 ± 0.77 |  | P = 1, vs. Bigger than Now  P = 0.047, vs. Smaller than Now |
| Bigger | 0.46 ± 0.85 |  | P = 0.114, vs. Smaller than Now |
| Smaller | 1.2 ± 0.86 |  |  |
| Satisfaction with Breast Size [range: 0-5] |  | P < 0.001 |  |
| About the Same | 4.4 ± 0.8 |  | P = 0.002*, vs. Bigger than Now  P < 0.001*, vs. Smaller than Now |
| Bigger | 2.8 ± 1.3 |  | P = 1, vs. Smaller than Now |
| Smaller | 2.4 ± 1.3 |  |  |
| Satisfaction with Weight [range: 0-5] |  | P = 0.013 |  |
| About the Same | 2.8 ± 1.3 |  | P = 0.049, vs. Bigger than Now  P = 1, vs. Smaller than Now |
| Bigger | 3.9 ± 1.3 |  | P = 0.02, vs. Smaller than Now |
| Smaller | 2.5 ± 0.7 |  |  |
| ASIR Composite [range: 1-5] |  | P = 0.53 | -- |
| About the Same | 3.1 ± 0.8 |  |  |
| Bigger | 3.2 ± 0.7 |  |  |
| Smaller | 3.3 ± 0.5 |  |  |
| BSI Global [range: 0-72] |  | P = 0.39 | -- |
| About the Same | 6.0 ± 4.8 |  |  |
| Bigger | 8.6 ± 9.5 |  |  |
| Smaller | 11.0 ± 9.5 |  |  |
| BIS [range: 0-30] |  | P = 0.28 | -- |
| About the Same | 4.68 ± 6.66 |  |  |
| Bigger | 6.00 ± 5.60 |  |  |
| Smaller | 8.40 ± 8.09 |  |  |
| BREAST-Q SWB [range: 0-100] |  | P = 0.015 |  |
| About the Same | 65.8 ± 24.9 | About the Same vs. Bigger than Now: | P = 0.147 |
| Bigger | 52.8 ± 19.3 | About the Same vs. Smaller than Now: | P = 0.035 |
| Smaller | 44.5 ± 17.4 | Bigger than Now vs. Smaller than Now: | P = 1 |
| BREAST-Q PSWB [range: 0-100] |  | 0.08 | -- |
| About the Same | 75.5 ± 24.8 |  |  |
| Bigger | 61.5 ± 14.6 |  |  |
| Smaller | 67.3 ± 22.6 |  |  |

*: significant with Bonferroni correction, †: Chi-square test

Supplementary Table 2. Univariate Multinomial Logistic Regression Models

| Variable | Preference | P value | Odds Ratio (95% CI) | Type III P value |
| --- | --- | --- | --- | --- |
| Age | BTN | 0.219 | 0.95 (0.88 – 1.02) | 0.29 |
|  | STN | 0.209 | 0.95 (0.86 – 1.03) |  |
| BMI | BTN | 0.037 | 0.803 (0.65 – 0.98) | 0.005 |
|  | STN | 0.111 | 1.16 (0.96 – 1.39) |  |
| Relationship Status (ref: in) | BTN | 0.48 | 0.43 (0.04 – 4.377) | 0.368 |
|  | STN | 0.35 | 2.25 (0.40 – 12.6) |  |
| Average Breast Volume | BTN | 0.003 | 0.99 (0.98 – 0.99) | <0.001 |
|  | STN | 0.13 | 1.00 (0.99 – 1.00) |  |
| Average Ptosis | BTN | 0.94 | 0.96 (0.37 – 2.46) | 0.067 |
|  | STN | 0.038 | 2.50 (1.05 – 5.95) |  |
| BIS | BTN | 0.53 | 1.04 (0.93 – 1.15) | 0.34 |
|  | STN | 0.15 | 1.08 (0.97 – 1.21) |  |
| Satisfaction with Breast Size | BTN | 0.0013 | 0.29 (0.14 - 0.62) | <0.001 |
|  | STN | 0.0005 | 0.21 (0.08 – 0.50) |  |
| Satisfaction with Weight | BTN | 0.017 | 2.24 (1.15 – 4.34) | 0.009 |
|  | STN | 0.45 | 0.78 (0.41 – 1.48) |  |
| BREAST-Q PSWB | BTN | 0.07 | 0.97 (0.94 – 1.00) | 0.158 |
|  | STN | 0.3 | 0.98 (0.95 – 1.02) |  |
| BREAST-Q SWB | BTN | 0.102 | 0.97 (0.94 – 1.00) | 0.023 |
|  | STN | 0.023 | 0.95 (0.91 – 0.99) |  |
| ASIR Composite | BTN | 0.67 | 1.24 (0.46 – 3.32) | 0.54 |
|  | STN | 0.28 | 1.84 (0.61 – 5.52) |  |
| BSI Global | BTN | 0.25 | 1.06 (0.96 - 1.18) | 0.175 |
|  | STN | 0.085 | 1.10 (0.99 – 1.22) |  |
| Reference preference group: About the Same  BTN: Bigger than Now Preference Group, STN: Smaller than Now Preference Group | | | | |

Supplementary Table 3. Three-covariate Multinomial Logistic Regression Model.

| Variable | Preference | Odds Ratio (95% CI) | P value | Type III P value |
| --- | --- | --- | --- | --- |
| Average Breast Volume | BTN | 0.995 (0.989-0.999) | 0.042 | 0.001 |
| Average Breast Volume | STN | 1.002 (0.999-1.005) | 0.264 |  |
| Satisfaction with Breast Size | BTN | 0.236 (0.083-0.666) | 0.006 | <0.001 |
| Satisfaction with Breast Size | STN | 0.144 (0.044-0.471) | 0.002 |  |
| BSI Global | BTN | 1.178 (0.967-1.435) | 0.103 | 0.09 |
| BSI Global | STN | 1.188 (0.959-1.473) | 0.115 |  |
| Reference preference group: About the Same  BTN: Bigger than Now Preference Group, STN: Smaller than Now Preference Group  AIC: 67.07, Hosmer-Lemeshow Test: p = 0.36 | | | | |

Supplementary Table 4. Four-covariate Multinomial Logistic Regression Model

| Variable | Preference | Odds Ratio (95% CI) | P value | Type III P value |
| --- | --- | --- | --- | --- |
| Average Breast Volume | BTN | 0.994 (0.989-0.999) | 0.043 | <0.001 |
| Average Breast Volume | STN | 1.002 (0.999-1.005) | 0.159 |  |
| Satisfaction with Breast Size | BTN | 0.238 (0.082-0.686) | 0.008 | <0.001 |
| Satisfaction with Breast Size | STN | 0.075 (0.014-0.396) | 0.002 |  |
| BSI Global | BTN | 1.186 (0.964-1.459) | 0.106 | 0.036 |
| BSI Global | STN | 1.332 (1.012-1.753) | 0.041 |  |
| BREAST-Q PSWB | BTN | 0.989 (0.949-1.030) | 0.584 | 0.109 |
| BREAST-Q PSWB | STN | 1.075 (0.976-1.183) | 0.141 |  |
| Reference preference group: About the Same  BTN: Bigger than Now Preference Group, STN: Smaller than Now Preference Group  AIC: 66.65, Hosmer-Lemeshow Test: p = 0.03 | | | | |

Supplementary Table 5. R Packages Used for Statistical Analysis

| Package Name | Reference |
| --- | --- |
| nnet | Venables, W. N. & Ripley, B. D. (2002) Modern Applied Statistics with S. Fourth Edition. Springer, New York. ISBN 0-387-95457-0. URL: http://www.stats.ox.ac.uk/pub/MASS4 |
| car | John Fox and Sanford Weisberg (2019). An {R} Companion to Applied Regression, Third Edition. Thousand Oaks CA: Sage. URL: https://socialsciences.mcmaster.ca/jfox/Books/Companion/ |
| generalhoslem | Matthew Jay (2019). generalhoslem: Goodness of Fit Tests for Logistic Regression Models. R package version 1.3.4. URL: https://CRAN.R-project.org/package=generalhoslem |
| stats | R Core Team (2019). R: A language and environment for statistical computing. R Foundation for Statistical Computing, Vienna, Austria. URL https://www.R-project.org/. |
| tidyverse | Wickham et al., (2019). Welcome to the tidyverse. Journal of Open Source Software, 4(43), 1686, https://doi.org/10.21105/joss.01686 |
